# The selective glucocorticoid receptor antagonist CORT125281 has tissue-specific activity

**DOI:** 10.1530/JOE-19-0486

**Published:** 2020-05-04

**Authors:** Lisa L Koorneef, Jan Kroon, Eva M G Viho, Lucas F Wahl, Kim M L Heckmans, Marloes M A R van Dorst, Menno Hoekstra, René Houtman, Hazel Hunt, Onno C Meijer

**Affiliations:** 1 Division of Endocrinology, Department of Medicine, Leiden University Medical Center, Leiden, The Netherlands; 2 Einthoven Laboratory for Experimental Vascular Medicine, Leiden University Medical Center, Leiden, The Netherlands; 3 Division of BioTherapeutics, Leiden Academic Centre for Drug Research, Leiden University, Leiden, The Netherlands; 4 Pamgene International, Den Bosch, The Netherlands; 5 Corcept Therapeutics, Menlo Park, California, USA

**Keywords:** corticosterone, glucocorticoid receptor, GR antagonist, HPA-axis, coregulators, mifepristone, RU486, tissue-specificity

## Abstract

Glucocorticoids mediate numerous essential processes in the human body via binding to the glucocorticoid receptor (GR). Excessive GR signaling can cause disease, and GR antagonists can be used to treat many symptoms of glucocorticoid-induced pathology. The purpose of this study was to characterize the tissue-specific properties of the selective GR antagonist CORT125281. We evaluated the antagonistic effects of CORT125281 upon acute and subchronic corticosterone exposure in mice. In the acute corticosterone setting, hypothalamus-pituitary-adrenal-axis activity was investigated by measurement of basal- and stress-induced corticosterone levels, adrenocorticotropic hormone levels and pituitary proopiomelanocortin expression. GR signaling was evaluated by RT-PCR analysis of GR-responsive transcripts in liver, muscle, brown adipose tissue (BAT), white adipose tissue (WAT) and hippocampus. Pretreatment with a high dose of CORT125281 antagonized GR activity in a tissue-dependent manner. We observed complete inhibition of GR-induced target gene expression in the liver, partial blockade in muscle and BAT and no antagonism in WAT and hippocampus. Tissue distribution only partially explained the lack of effective antagonism. CORT125281 treatment did not disinhibit the hypothalamus-pituitary-adrenal neuroendocrine axis. In the subchronic corticosterone setting, CORT125281 partially prevented corticosterone-induced hyperinsulinemia, but not hyperlipidemia and immune suppression. In conclusion, CORT125281 antagonizes GR transcriptional activity in a tissue-dependent manner and improves corticosterone-induced hyperinsulinemia. Tailored dosing of CORT125281 may allow tissue-specific inhibition of GR transcriptional activity.

## Introduction

Glucocorticoids (GCs) are adrenal hormones involved in the stress response, regulating processes such as immune function and metabolism. The hypothalamus-pituitary-adrenal (HPA)-axis controls GC secretion via a cascade of hormonal events: corticotrophin-releasing hormone from the hypothalamic paraventricular nucleus (PVN) induces adrenocorticotropic hormone (ACTH) release by the anterior pituitary, thereby triggering GC production and secretion by the adrenals (Herman *et al.* 2016). The most widely expressed GC receptor is the glucocorticoid receptor (GR), which mediates many effects on target tissues while simultaneously dampening HPA-axis activity via negative feedback loops at the level of the pituitary and the PVN. Given the pleiotropic nature of GR signaling, excessive HPA-axis activity is accompanied by disease, as exemplified by patients with Cushing’s syndrome (Ferrau & Korbonits 2015). The classic GR antagonist mifepristone (RU486) is commonly used to treat these patients (Cuevas-Ramos *et al.* 2016). In research settings, mifepristone is often applied to experimentally manipulate GR activity. However, mifepristone also antagonizes the progesterone and androgen receptors (Jung-Testas & Baulieu 1983, Gaillard *et al.* 1984, Kloosterboer *et al.* 1995, Kroon *et al.* 2018) and can exhibit partial agonistic properties at GR (Havel *et al.* 1996, van den Heuvel *et al.* 2016, Kroon *et al.* 2018). Therefore, there is a need for more selective GR antagonists.

Tissue responsiveness to GR ligands depends on multiple levels of regulation and sensitivity can, therefore, be highly divergent. Besides plasma concentrations, bioavailability of GCs at the receptor depends on the activity of efflux pumps (Karssen *et al.* 2001, Nixon *et al.* 2016) and of steroid-converting enzymes in the liver and other target tissues (Morgan *et al.* 2016). Subsequent ligand-binding to GR leads to the translocation of the ligand-receptor complex to the nucleus where the complex can bind to the DNA to induce or repress gene expression, generally involving parallel regulation of hundreds of genes. Eventual GR transcriptional activity is influenced by ligand concentration, receptor levels and the presence of transcriptional coregulator proteins. As the factors that determine GR transcriptional output are highly context dependent, genomic GC action is strongly tissue specific. Likewise, the efficacy of GR antagonists can depend on context-specific pharmacokinetics and pharmacodynamic cellular characteristics.

CORT125281 is a novel GR antagonist that is being developed to counteract pathologies that result from overactive GC signaling (Hunt *et al.* 2017). Unlike mifepristone, CORT125281 lacks cross-reactivity with other nuclear receptors (Kroon *et al.* 2018). CORT125281 has beneficial effects in metabolic disease models, which is likely, in part, mediated via activation of brown adipose tissue (BAT) (Kroon *et al.* 2018). In this study, we characterized the antagonistic activity of CORT125281 in various GC-sensitive tissues in lean male mice. We show that GR antagonism by CORT125281 is not only dose dependent, but also strongly tissue dependent.

## Materials and methods

### Animals

All studies involving animals were approved by the institutional ethics committee on animal care and experimentation at the Leiden University Medical Center. The 8-week old male C57Bl/6J mice (Charles River) were housed in ventilated cages with a 12 h light:12 h darkness regime and *ad libitum* access to food and water. In the acute corticosterone exposure experiments, mice received a synthetic chow diet (Research Diets, New Brunswick, NJ, USA) containing vehicle or CORT125281 (6, 20 or 60 mg/kg/day, Corcept Therapeutics, Menlo Park, CA, USA) for 6 days. At day 6, mice were injected subcutaneously with 200 µL solvent (EtOH:PBS 1:20) or 1.5 mg/kg corticosterone (Sigma-Aldrich) 1 h prior to killing by cervical dislocation. In the subchronic corticosterone exposure experiment, standardized conditions were used, that is, regular chow diet, in which we evaluated a therapeutic treatment regimen of daily oral antagonist administration. Mice were subcutaneously implanted with a sham- (100 mg cholesterol) or a corticosterone-pellet (12.5 mg corticosterone (Sigma-Aldrich), 87.5 mg cholesterol) while under isoflurane anesthesia and buprenorphine analgesia (0.03 mg/kg, Reckitt Benckiser Healthcare, Hull, United Kingdom). Mice were treated daily with vehicle, 60 mg/kg mifepristone (Corcept Therapeutics) or 60 mg/kg CORT125281 (dissolved in PBS with 0.1% Tween 80 and 0.5% hydroxypropylmethylcellullose, Corcept Therapeutics) via oral gavage for 5 days, after which mice were killed by CO_2_ inhalation. Throughout the experiment, body weight was monitored and body composition was determined with EchoMRI™ (Frankfurt, Germany). After 5 days, blood was collected from the lateral tail vain in paraoxon-coated capillaries after a 6-h fast. Plasma triglyceride, total cholesterol, free fatty acid, glucose and insulin levels were determined using commercially available kits (Roche; Instruchemie, Delfzijl, Netherlands and Crystal Chem, Zaandam, Netherlands). Blood concentrations of the major leukocyte classes, that is, lymphocytes, monocytes and eosinophils, were determined in whole blood using an automatic veterinary hematology analysis (Sysmex XT-2000iV; Goffin Meyvis, Etten-Leur, the Netherlands).

In both acute and subchronic corticosterone experiments, relevant tissues (including quadriceps muscle, interscapular BAT, gonadal white adipose tissue (WAT), adrenals, spleen, thymus, pituitary and hippocampus) were collected to determine tissue weight and/or for further molecular analysis.

### ACTH and corticosterone measurements and novelty stress test

Stress-free blood samples were collected via a tail incision within 60 s for ACTH and within 120 s for corticosterone, that is, before hormone levels rise due to the sampling procedure. Samples for basal plasma corticosterone levels were collected at 08:00 h and 18:00 h at day 2 and 4. Novelty stress tests were performed in the morning (between 08:00–12:00 h) after 2 days of exposure to CORT125281. At t = 0, a blood sample was collected, after which mice were placed in a novel cage without bedding. After 10 min, a second blood sample was collected and mice were placed back in their original home cage. Blood was additionally collected at t = 30, 60 and 120 min. Plasma corticosterone and ACTH levels were determined using a HS EIA kit (Immunodiagnosticsystems, Boldon Business Park, UK) or a Double Antibody hACTH ^125^I RIA kit (MP Biomedicals, Amsterdam, Netherlands), respectively.

### RNA isolation, cDNA synthesis and real-time quantitative PCR

Total RNA was isolated from snap-frozen tissues using TriPure isolation reagent (Roche). After reverse transcription to cDNA (Promega), RT-qPCR was performed using IQ SYBR-Green supermix and MyIQ thermal cycler (Bio-RAD CFX96). Primers were tested for high efficiency (90–110%) and for amplification of a single PCR product. All primer sequences are listed in Supplementary Table 1 (see section on supplementary materials given at the end of this article), with the exception of *Ncor1* and *Ncor2* which were purchased from Qiagen. Fold change expression was calculated using the 2^−ΔΔ^*^CT^* method.

### *In situ* hybridization

For dual label *in situ* hybridization, frozen unfixed pituitaries were embedded in tissue-tek OCT (Sakura Finetek, Alphen aan den Rijn, Netherlands), sliced (12 µm) and collected on glass slides (Superfrost Plus, Thermo Fisher Scientific). Dual label *in situ* hybridization was performed using the QuantiGene ViewRNA ISH Tissue 2-Plex Assay (Thermo Fisher Scientific) and pictures were captured with a wide-field microscope (DM6B, Leica Microsystems). For 4-plex *in situ* hybridization, frozen, unfixed brain slices (12 µm) were collected on glass slides at bregma −0.772 mm (PVN) and −1.532 mm (hippocampus). 4-plex *in situ* hybridization was performed using the RNAScope fluorescent multiplex assay and RNAscope 4-plex ancillary kit (Advanced Cell Diagnostics, Newark, CA, USA) and Opal fluorophores 520, 570, 620 and 690 (PerkinElmer). Pictures were captured with a confocal microscope (SP8 WLL, Leica Microsystems). All pictures were analysed with ImageJ Software (NIH).

### Quantification of serum and tissue CORT125281 concentration

Compound levels were assessed against standard curves for CORT125281, ranging from 0.05–20 µM in each tissue. Tissues were weighed and water was added at three-fold the weight of each individual sample before homogenising. Internal standard (90 µL of 1 µM leucine enkephalin in acetonitrile) was added to all samples, standard curves and quality control spikes of CORT125281. After centrifugation, a fixed volume of supernatant was removed and pellets were dissolved in water. The samples were then analysed by LC-MS utilising a QTRAP 5500 mass spectrometer (Sciex, Warrington, UK), UPLC (Agilent) instrument, equipped with a C18 Cortecs UPLC analytical column (2.1 × 30 mm, 1.6 mm particle size). Separation was achieved with a time programmed gradient of mobile phase A (water + 0.1% (v/v) formic acid) and mobile phase B (acetonitrile + 0.1% (v/v) formic acid) with a flow rate of 0.65 mL/min at a temperature of 35°C. The gradient started with a mobile phase composition of 5% B which was held for 0.1 min. The percentage of B was then linearly ramped to 95% over 0.1 to 1.2 min and then held constant up to 1.3 min. Finally, the column was re-equilibrated for 0.1 min at the initial conditions of 5% B. The retention time of CORT125281 was 0.86 min. The mass spectrometer was set to operate in negative ion mode. The ion source temperature was 650°C, the ion spray voltage was −4000 V, gases 1, 2 and curtain gas were 30 V, 60 V and 35 V, respectively, and the entrance and collisition exit potential were −10 V and −13 V. The collision energy and declustering potential used for the multiple reaction monitoring (MRM) transitions of CORT125281 were −28 V and −60 V, the Q1 and Q3 masses were 588.649 Da and 248.800 Da, while the collision energy and declustering potential for the internal standard were −40 V and 0 V and the Q1 and Q3 masses 554.2 Da and 235.9 Da.

### Peptide interaction profiling

Interactions between the GR ligand-binding domain and coregulator nuclear receptor boxes were determined by MARCoNI (microarray assay for real-time coregulator-nuclear receptor interaction), as described previously (Koppen *et al.* 2009, Desmet *et al.* 2014). As the assay utilizes human GR, experiments were performed with cortisol rather than corticosterone. In brief, each array containing a set of 154 peptides representing coregulator-derived nuclear receptor binding motifs was incubated with a reaction mixture of 1 nM glutathione *S*-transferase (GST), tagged GR-ligand-binding domains (PV4689), Alexa Fluor 488-conjugated GST antibody (25 nM, A-11131), buffer F (PV4547; all Invitrogen) and vehicle (2% DMSO in water) with or without receptor ligands cortisol (1 µM), dexamethasone (1 µM), mifepristone (0.1 µM) or CORT125281 (1 µM). Incubation was performed at 20°C in a PamStation96 (PamGene International, Den Bosch, Netherlands) for 40 min. Receptor binding to each peptide on the array, reflected by a fluorescent signal, was quantified by analysis of .tiff images using BioNavigator software (PamGene International).

### Statistical analysis

All data are expressed as mean ± s.e.m. All *P*-values are two-tailed and *P* < 0.05 was considered as statistically significant; *P* < 0.1 as statistical trend. Balanced data concerning more than two groups with one or two factors were analysed with a one-way or two-way ANOVA, respectively, with a Tukey post hoc test. Unbalanced data with two factors were analysed with linear mixed models, which included independent variables as fixed factors and dependent variables as random factors. For the random effects, that is, random slopes and intercepts, the model used an unstructured covariance matrix. The main parameters for our power calculations were based on gene expression of GR target genes, with a power of 80%, type I error probability of 0.05, a difference of 40% for the 60 mg/kg/day dose and of 30% for the 6 mg/kg/day and a s.d. of ~20–25%.

## Results

### CORT125281 inhibits GR transcriptional activity in a tissue-dependent manner

**Figure 1:**
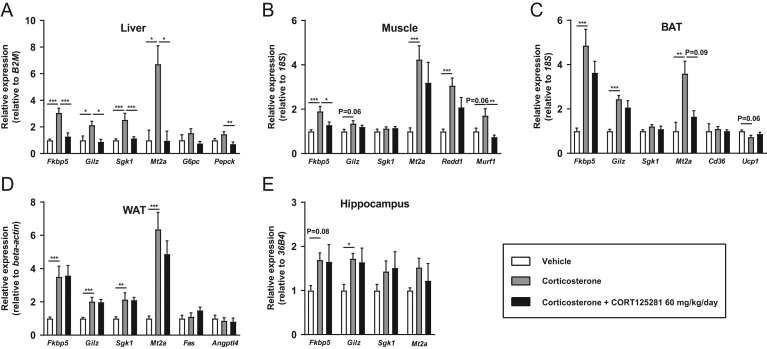
CORT125281 exhibits tissue-specific GR antagonism. Mice received vehicle or 60 mg/kg/day CORT125281 for 6 days and received a solvent or corticosterone injection 1 h prior to killing. (A) In the liver, CORT125281 fully blocked the corticosterone-induced upregulation of general GR target genes *Fkbp5*, *Gilz*, *Sgk1* and *Mt2a* and liver-specific GR target genes *G6pc* and *Pepck*. CORT125281 inhibited a subset of genes in (B) quadriceps muscle and (C) brown adipose tissue (BAT) and did not show efficacy in (D) white adipose tissue (WAT) and (E) hippocampus. Values are means ± s.e.m. of *n* = 7 mice per group. Statistical significance was calculated using a one-way ANOVA with Tukey’s multiple comparisons test. *P < 0.05, **P < 0.01, ***P < 0.001.

We first evaluated the overall capacity of CORT125281 to inhibit GR transcriptional activity by acute injection of corticosterone (1.5 mg/kg) or solvent in animals pretreated with a high dose of CORT125281 (60 mg/kg/day, via the food). CORT125281 treatment did not alter body weight nor food intake (Supplementary Fig. 1A, B and C). Using RT-qPCR, we analyzed in several tissues expression levels of four general and well-established GR target genes: FK506-binding protein 51 (*Fkbp5*), glucocorticoid-induced leucine zipper (*Gilz*/*Tsc22d3*), Serum/Glucocorticoid Regulated Kinase 1 (*Sgk1*) and Metallothionein 2 (*Mt2a*). Corticosterone upregulated expression of all GR target genes in liver, muscle, BAT, WAT and hippocampus, with the exception of *Sgk1* in muscle, BAT and hippocampus and *Mt2a* in hippocampus (Fig. 1A, B, C, D and E). We observed that CORT125281 exhibited a tissue-specific pattern of GR antagonism: that is, complete blockade of corticosterone-induced mRNA in the liver (Fig. 1A); inhibition of only a subset of corticosterone-induced genes in muscle and BAT (Fig. 1B and C) and no effects in WAT and hippocampus (Fig. 1D and E). In the liver, CORT125281 also blocked the induction of tissue-specific GR target gene phosphoenolpyruvate carboxykinase 1 (*Pepck*), and a similar pattern was observed for glucose-6-phosphatase (*G6pc*) (Imai *et al*. 1990, Vander Kooi *et al*. 2005). Ubiquitin E3 ligase (*Murf1*) induction was completely blocked in muscle, unlike protein regulated in development and DNA damage response 1 (*Redd1*) (Waddell *et al.* 2008, Shimizu *et al.* 2011). In white adipose tissue, *Fas* and *Angptl4* were not induced by corticosterone in this time frame. We separately studied the effects of CORT125281 treatment (60 mg/kg/day) in intact animals without prior corticosterone exposure. CORT125281 inhibited mRNA levels of *Gilz* and *G6p* in the liver (Supplementary Fig. 1D) but did not affect GR target gene expression in muscle and WAT (Supplementary Fig. 1E and F). CORT125281 upregulated *Cd36* in BAT while it downregulated expression of *Sgk1* and *Mt2a* in the hippocampus, albeit, to a very modest extent (Supplementary Fig. 1G and H). Collectively the data show that CORT125281 inhibits GR transcriptional activity in a tissue-specific manner, both after corticosterone injection and under basal conditions.

### CORT125281 inhibits GR transcriptional activity in a dose-dependent manner in peripheral tissues

**Figure 2:**
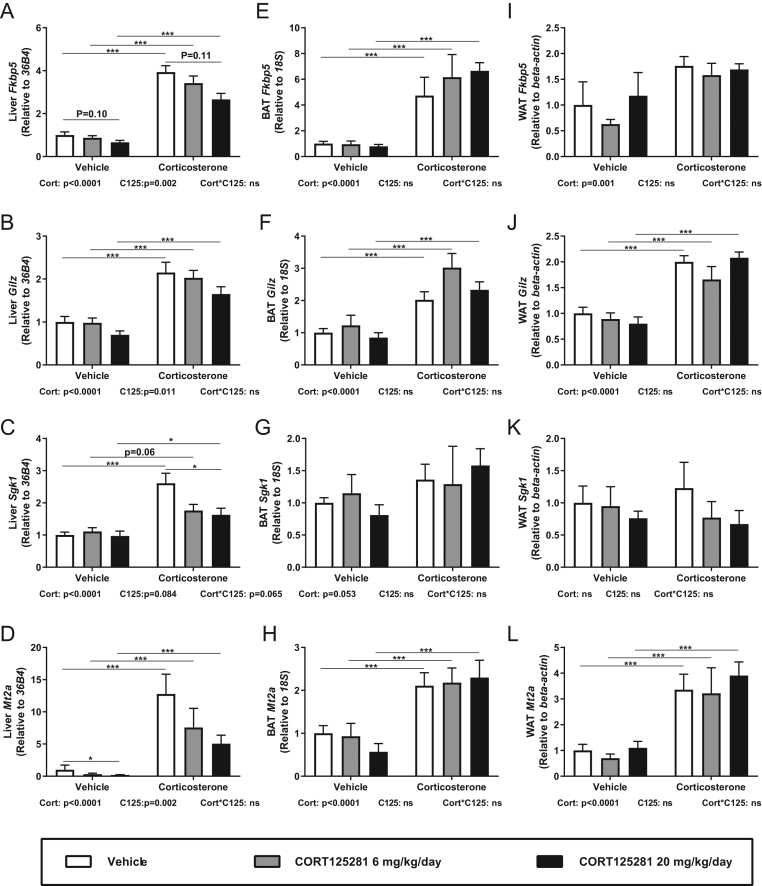
CORT125281 inhibits GR transcriptional activity in a dose- and tissue-dependent manner. Mice received vehicle or 6–20 mg/kg/day CORT125281 for 6 days and received a solvent or corticosterone injection 1 h prior to killing. CORT125281 partially prevented the corticosterone-induced upregulation of (A, B, C and D) *Fkbp5*,* Gilz*, *Sgk1* and *Mt2a* in the liver. CORT125281 did not antagonize GR transcriptional activity in (E, F, G and H) brown adipose tissue (BAT) and (I, J, K and L) white adipose tissue (WAT). Values are means ± s.e.m. of *n* = 7–8 mice per group. Statistical significance was calculated using a two-way ANOVA with Tukey’s multiple comparisons test. *P < 0.05, ***P < 0.001.

To characterize dose dependency of GR antagonism, we performed a study with lower doses of CORT125281 (6 and 20 mg/kg/day) for 6 days before corticosterone or solvent injection 1 h prior to tissue collection. CORT125281 treatment again did not alter body weight or food intake (Supplementary Fig. 2A, B and C). As expected, we observed a robust upregulation of most GR-responsive genes after corticosterone treatment (Fig. 2A, B, C, D, E, F, G, H, I, J and L). In the liver, CORT125281 administration at lower doses partially inhibited corticosterone-induced GR transcriptional activity of *Fkbp5*, *Gilz, Sgk1* and *Mt2a* (Fig. 2A, B, C and D). CORT125281 also inhibited *Mt2a* expression in solvent-injected mice (Fig. 2D). For certain GR-responsive genes, 6 mg/kg/day CORT125281 was sufficient to attenuate corticosterone-induced expression (i.e. *Sgk1* and *Mt2a*, Fig. 2C and D), while for others a dose of 20 mg/kg/day was required (i.e. *Fkbp5* and *Gilz*, Fig. 2A and B). The potency of CORT125281 for effective inhibition thus differed per GR target gene, conform Monczor et al. (Monczor *et al.* 2019). In BAT, the antagonistic actions of CORT125281 were completely lost at the 6–20 mg/kg/day doses (Fig. 2E, F, G and H). Similar to the 60 mg/kg/day CORT125281 results, lower doses of CORT125281 did not inhibit GR transcriptional activity in WAT, even though the induction by corticosterone in this tissue tended to be modest in this experiment (Fig. 2I, J, K and L).

### CORT125281 does not antagonize GR activity in the brain during excess corticosterone exposure

**Figure 3:**
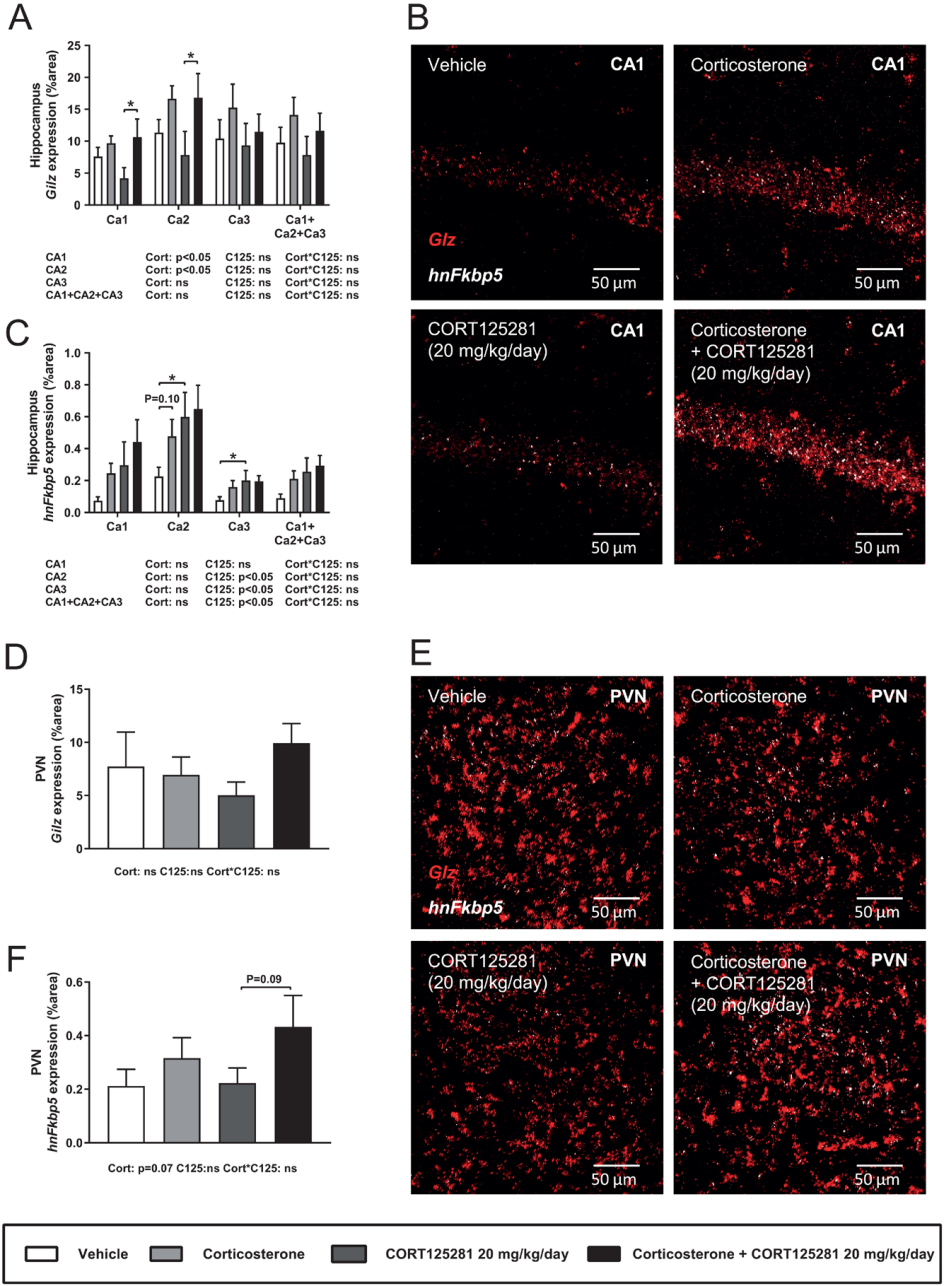
CORT125281 does not antagonize *Gilz* and *Fkbp5* expression in the brain during excess corticosterone exposure. Mice received vehicle or 20 mg/kg/day CORT125281 for 6 days and received a vehicle or corticosterone injection 1 h prior to killing. (A, B and C) Corticosterone increased expression levels of *Gilz* (red) mRNA in CA1 and CA2 regions of the hippocampus and of *Fkbp5* hnRNA (white) in the CA2, which was not prevented by CORT125281. (D, E and F) In the paraventricular nucleus of the hypothalamus (PVN), corticosterone did not affect *Gilz* (red) expression, but tended to induce *Fkbp5* (white) hnRNA expression, which was not prevented by CORT125281. Values are means ± s.e.m. of *n* = 7–8 mice per group. Statistical significance was calculated using linear mixed models. *P < 0.05. A full colour version of this figure is available at https://doi.org/10.1530/JOE-19-0486.

**Figure 4:**
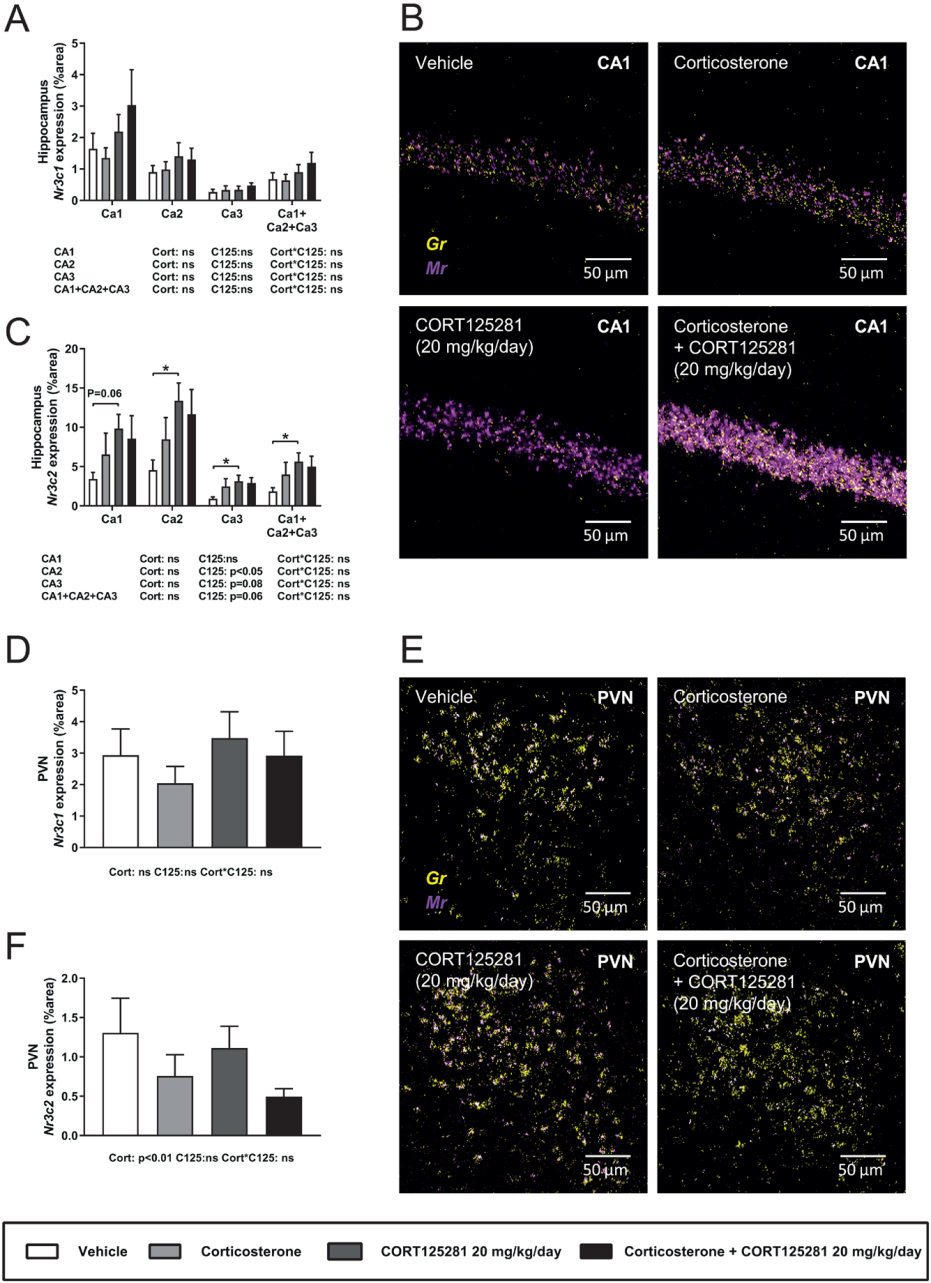
CORT125281 does not inhibit corticosterone-induced *Nr3c1* and *Nr3c2* expression in the brain. Mice received vehicle or 20 mg/kg/day CORT125281 for 6 days and received a vehicle or corticosterone injection 1 h prior to killing. (A, B and C) Neither corticosterone nor CORT125281 affected *Nr3c1* (*Gr*, yellow) mRNA levels in the hippocampus, but CORT125281 upregulated *Nr3c2* (*Mr*, magenta) mRNA levels in all regions of the hippocampus. (J, K and L) Neither corticosterone nor CORT125281 affected *Nr3c1* (yellow) *or Nr3c2* (magenta) expression in the paraventricular nucleus of the hypothalamus (PVN). Values are means ± s.e.m. of *n* = 7–8 mice per group. Statistical significance was calculated using linear mixed models. *P < 0.05. A full colour version of this figure is available at https://doi.org/10.1530/JOE-19-0486.

To analyze activity of CORT125281 in the brain at a cellular level, we measured *Gilz* mRNA and *Fkbp5* heteronuclear RNA (hnRNA) levels in the hippocampus and PVN by *in situ* hybridization. Mice were treated with CORT125281 (6–20 mg/kg/day) or vehicle and received a corticosterone or solvent injection 1 h prior to tissue collection. We chose hnRNA, since hippocampal *Fkbp5* hnRNA levels peak 1 h after corticosterone exposure while *Fkbp5* mRNA levels peak after 3 h (Mifsud & Reul 2016). Corticosterone upregulated *Gilz* mRNA and *Fkbp5* hnRNA in most hippocampal subfields, while CORT125281 did not prevent this induction (Fig. 3A, B and C). However, CORT125281 upregulated basal levels of *Fkbp5* hnRNA in CA2 and CA3 regions (Fig. 3C). In the PVN, corticosterone increased *Fkbp5* hnRNA, but not *Gilz* mRNA levels (Fig. 3D, E and F). Similar to the hippocampus, CORT125281 had little effect on corticosterone-induced *Fkbp5* hnRNA levels and *Gilz* mRNA levels in the PVN. Hippocampal expression of GR (*Nr3c1*) mRNA was unaltered by all treatments (Fig. 4A and B), CORT125281 increased basal mineralocorticoid receptor (MR; *Nr3c2*) mRNA expression in the hippocampus (Fig. 4B and C) and in the PVN and mRNA expression of both receptors was unaffected by all treatments (Fig. 4D, E and F). In summary, 6 and 20 mg/kg CORT125281 did not inhibit corticosterone-induced mRNA expression in the hippocampus and PVN, but did upregulate *hnFkbp5* and *Nr3c2* expression in the hippocampus.

### CORT125281 does not interfere with GR-mediated negative feedback

**Figure 5:**
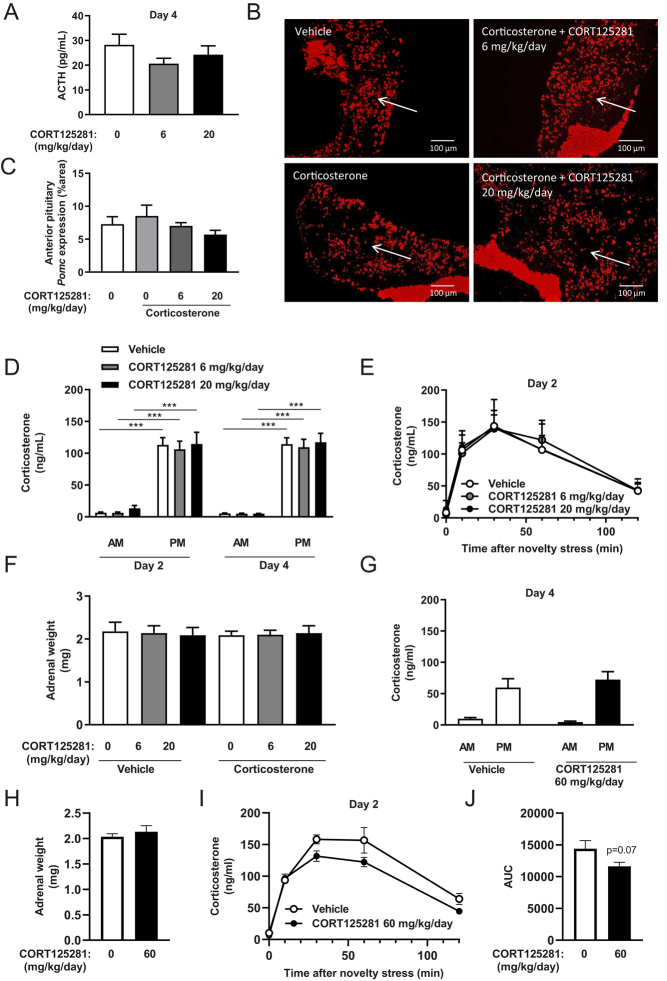
CORT125281 does not influence basal- or stress-induced HPA-axis activity. Mice received vehicle or 6–20 mg/kg/day CORT125281 for 6 days and received a solvent or corticosterone injection 1 h prior to killing. (A) CORT125281 did not alter basal ACTH levels at 18:00 h. Statistical significance was calculated with a one-way ANOVA with Tukey’s multiple comparisons test. (B and C) CORT125281 did not affect *Pomc* expression (red) in the anterior pituitary (white arrows). (D) CORT125281 did not influence basal corticosterone levels at the trough (08:00 h) and peak (18:00 h). Statistical significance was calculated using linear mixed models. (E) CORT125281 did not alter novelty stress-induced corticosterone levels at day 2. (F) Weight of GC-responsive adrenals was unaltered upon CORT125281 treatment. Statistical significance was calculated with a two-way ANOVA with Tukey’s multiple comparisons test. (G and H) In mice treated with vehicle or 60 mg/kg/day CORT125281, CORT125281 did not alter basal corticosterone levels at the trough (08:00 h) and peak (18:00 h). Statistical significance was calculated with a two-way ANOVA. (H, I and J) While adrenal weights were unaltered by CORT125281 treatment, peak corticosterone levels after a novelty stressor and the area under the curve (AUC) tended to be reduced. Statistical significance was calculated with an independent sample *t*-test. Values are means ± s.e.m. of *n* = 6–8 per group. *** *P* < 0.001. A full colour version of this figure is available at https://doi.org/10.1530/JOE-19-0486.

Many GR antagonists display some partial agonistic effects on the GR, thereby affecting HPA-axis activity *in vivo*. We previously showed that chronic administration of mifepristone but not CORT12581 via the food suppressed endogenous corticosterone levels (Kroon *et al.* 2018). In fact, CORT125281 restored the diurnal corticosterone rhythm which was flattened by the high-fat diet in these experiments (Kroon *et al.* 2018). In the current study, we investigated the influence of continuous CORT125281 treatment on HPA-axis activity and corticosterone rhythm under regular diet conditions. After 2 and 4 days of CORT125281 administration (6–20 mg/kg/day), we collected stress-free blood for hormone measurement at trough (08:00 h) and peak (18:00 h). At 18:00 on day 4, circulating levels of ACTH were unchanged by CORT125281 treatment at both dosages (Fig. 5A), which was in line with unaltered expression of pro-opiomelanocortin (*Pomc*) in the anterior pituitary, which after enzymatic cleavage yields the ACTH peptide (Fig. 5B and C). The anterior pituitary was responsive to CORT125281 treatment though, as GR (*Nr3c1*) mRNA expression was increased by CORT125281 treatment (Supplementary Fig. 3A, B and C). Of note, GR expression was considerably higher in the corticotropes compared to the rest of the anterior pituitary (Supplementary Fig. 3D). CORT125281 did not alter basal peak or trough corticosterone levels (Fig. 5D). As GR antagonists can interfere with negative feedback and thereby disinhibit the HPA-axis (Gaillard *et al.* 1984, De Kloet *et al.* 1988), we further examined HPA-axis activity upon novelty stress. At all measured time points after the novelty stress, corticosterone levels were similar between vehicle, 6 and 20 mg/kg/day CORT125281-treated mice (Fig. 5E), as were the adrenal weights (Fig. 5F). The lack of effects of CORT125281 could be explained by the lower dose used in this experiment. We, therefore, analyzed basal and stress-induced HPA-axis activity in mice treated with 60 mg/kg/day CORT125281. Also at a higher dose, CORT125281 did not alter peak and trough corticosterone levels nor adrenal weights (Fig. 5G and H). Peak novelty stress-induced corticosterone levels were slightly attenuated (Fig. 5I and J), while the slope during the recovery phase (between t = 30 and 120 min) was unaltered. Altogether, these results demonstrate that CORT125281 does not suppress or disinhibit basal and stress-induced corticosterone levels.

### CORT125281 tissue-distribution or coregulator recruitment does not fully explain tissue-specific GR antagonism

**Figure 6:**
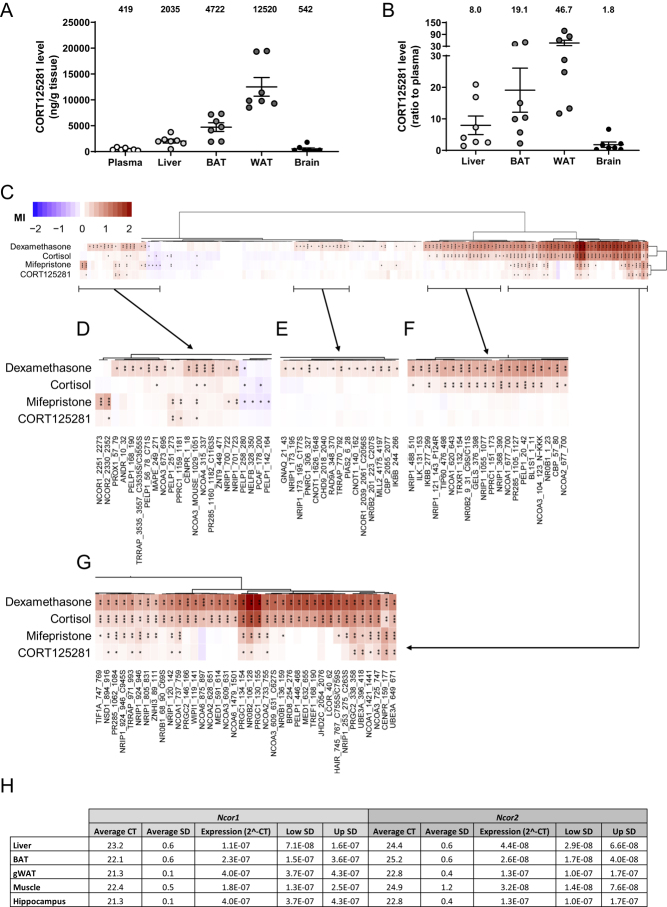
CORT125281 tissue-distribution or coregulator recruitment does not fully explain tissue-specific GR antagonism. Mice received 60 mg/kg/day CORT125281 for 6 days and plasma, liver, brown adipose tissue (BAT), gonadal white adipose tissue (gWAT) and the neocortex of the brain were collected to determine CORT125281 concentrations via mass spectrometry. Concentrations are expressed as (A) absolute tissue levels and as (B) tissue to plasma ratio. Values are means ± s.e.m. of *n* = 7 mice per group. (C) To investigate which coregulators were recruited by CORT125281, the ligand-induced interactions with coregulator motifs were identified with a MARcoNI assay (microarray assay for real-time coregulator-nuclear receptor interaction) and quantified as modulation index (MI, log fold change relative to vehicle). The heatmap shows hierarchically clustered modulation indexes for the applied ligands dexamethasone (top row), cortisol (second row), mifepristone (third row) and CORT125281 (bottom row). Stars indicate significance relative to vehicle. Some clusters are shown in more detail; (D) Cluster which includes corepressors NCOR1 and -2. (E) Motifs exclusively bound by the dexamethasone-GR complex. (F) Motifs for which interactions are induced by both dexamethasone and cortisol. (G) Motifs for which interactions are induced by all ligands. (H) To explain the tissue-specific actions of CORT125281, *Ncor1* and *2* expression was determined in liver, BAT, WAT, quadriceps muscle and hippocampus in vehicle-treated animals. Values are means ± s.e.m. of *n* = 4–6 mice per group. A full colour version of this figure is available at https://doi.org/10.1530/JOE-19-0486.

To examine if tissue-specific patterns of GR antagonism could be explained by differences in exposure, we measured CORT125281 concentrations in plasma, liver, BAT, WAT and the brain neocortex of mice treated with 60 mg/kg/day CORT125281 (Fig. 6A and B). High CORT125281 levels were observed in WAT (46.7 tissue to plasma ratio), intermediate levels were observed in liver and BAT (8.0 and 19.1 tissue to plasma ratio, respectively) and low levels were detected in the brain (1.8 tissue to plasma ratio). For the brain, the lack of GR antagonism may thus be explained by inadequate penetrance of CORT125281. Interestingly, hepatic GR activity was fully inhibited upon CORT125281 treatment despite relatively modest tissue concentrations. For WAT, alternative explanations are required, as the high tissue levels of CORT125281 were not accompanied by GR antagonism. One possibility is that CORT125281 requires the interaction between the GR and particular transcriptional corepressors for adequate antagonism, which may be expressed in a tissue-specific manner (Schoch *et al.* 2010). We, therefore, compared the coregulator recruitment by GR for CORT125281, the GR antagonist mifepristone and GR agonists cortisol and dexamethasone (Fig. 6C). Hierarchal clustering of ligand-induced GR-coregulator interactions revealed that the CORT125281 response profile clustered with that of mifepristone. The two antagonists differed in that the corepressor motifs of NCOR1 and NCOR2 (also known as SMRT) were strongly bound by the mifepristone-GR complex, but only very modestly by CORT125281-GR (Fig. 6D). In general, the ligand-induced interactions followed ‘classic’ pharmacology at the majority of coregulator motifs: that is, GR agonists dexamethasone and cortisol induced motif binding, while both GR antagonists lacked this effect (Fig. 6E and F). Of note, dexamethasone induced substantial interactions for a number of motifs where cortisol was without effect (Fig. 6E). A number of motifs were bound by all ligand-GR complexes, although the interactions induced by GR antagonists were usually weaker as compared to the GR agonists (Fig. 6D and G). CORT125281 tended to recruit fewer motifs compared to mifepristone (Fig. 6G). Given that CORT125281- and RU486-GR complexes differentially recruited the corepressors NCOR1- and 2, we hypothesized that differential *Ncor1/2* tissue expression may explain the different actions of RU486 and CORT125281. Using RT-qPCR, *Ncor1* levels were found to be 2–4 times lower in liver compared to other tissues, and *Ncor2* expression was also 2–4 times lower in liver than in WAT and hippocampus (Fig. 6H). The data show that lack of antagonism by CORT125281 tends to be associated with high levels of corepressor expression.

### CORT125281 prevents corticosterone-induced hyperinsulinemia

**Figure 7:**
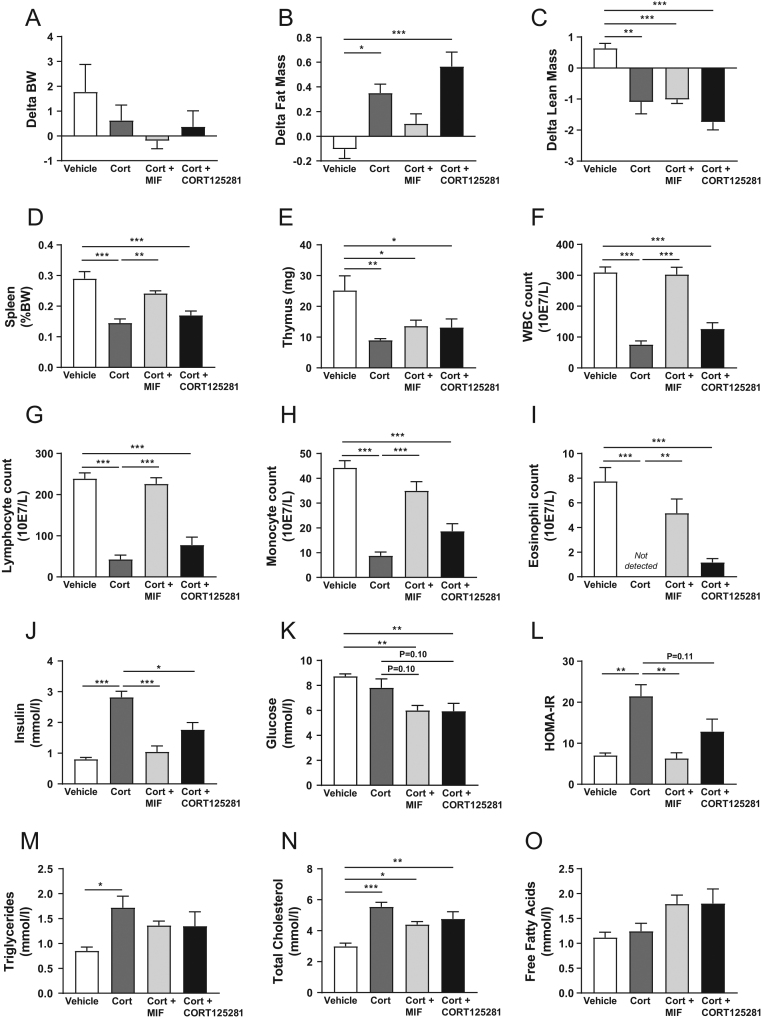
CORT125281 prevents subchronic corticosterone-induced hyperinsulinemia. Mice were exposed to sham or corticosterone (Cort) pellets for 5 days and were treated with CORT125281 (60 mg/kg/day) or mifepristone (MIF, 60 mg/kg/day) via oral gavage. CORT125281 did not prevent corticosterone-induced alterations in body weight (BW) gain (A), fat mass (B), lean mass (C), spleen weight (D), thymus weight (E), total white blood cells (F) (WBC), lymphocyte count (G), monocyte count (H) and eosinophil count (I). CORT125281 partially and mifepristone fully reversed the corticosterone-induced increase in (J) insulin levels, which resulted in improved (K) glucose levels and (L) HOMA-IR. Both CORT125281 and mifepristone seemed to modestly reduce (M) plasma triglycerides, total cholesterol levels (N), but did not affect (O) free fatty acid levels. Values are expressed as means ± s.e.m. of *n* = 4–6 mice per group. Statistical significance was calculated using one-way ANOVA with Tukey’s multiple comparisons test. *P < 0.05, **P < 0.01, ***P < 0.001.

**Figure 8:**
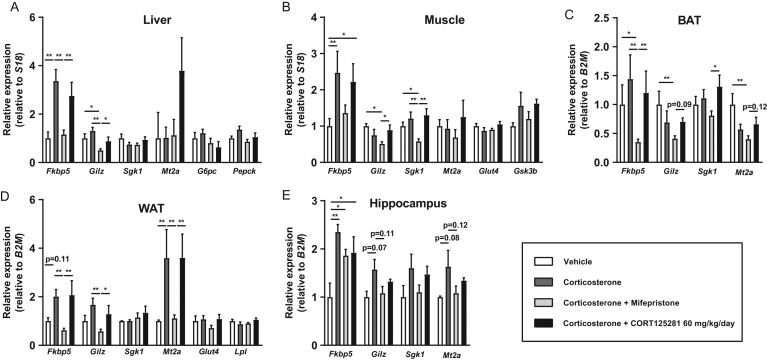
CORT125281 does not inhibit subchronic corticosterone-induced GR transcriptional activity. Mice were exposed to vehicle- or corticosterone-pellets for 5 days and were treated with CORT125281 (60 mg/kg/day) or mifepristone (60 mg/kg/day) via oral gavage. CORT125281 did not prevent the corticosterone-induced upregulation of GR target genes in (A) liver, quadriceps muscle (B), brown adipose tissue (C) (BAT), white adipose tissue (D) (WAT) and (E) the hippocampus. CORT125281 additionally did not affect expression of insulin target genes *G6p* and *Pepck* in liver, *Glut4* and *Lpl* in WAT and *Glut4* and *Gsk3b* in muscle. Values are means ± s.e.m. of *n* = 4–6 mice per group. Statistical significance was calculated using a one-way ANOVA with Tukey’s multiple comparisons test. *P < 0.05, **P < 0.01, ***P < 0.001.

We next tested the ability of CORT125281 to reverse adverse metabolic effects of subchronic corticosterone exposure and compared this head-to-head with mifepristone. Upon corticosterone exposure, mice increased in body weight due to an increase in fat mass (Fig. 7A and B). Fat mass was non-significantly reduced by mifepristone, but not by CORT125281 (Fig. 7B). Lean mass was reduced upon subchronic corticosterone exposure and was not altered by either mifepristone or CORT125281 treatment (Fig. 7C). Corticosterone decreased the spleen weight (Fig 7D), which was prevented by mifepristone but not CORT125281, while thymus weight was reduced in all corticosterone-exposed groups (Fig. 7E). Mifepristone but not CORT125281 fully prevented the corticosterone-induced immune suppression, that is, the cell count of total white blood cells, lymphocytes, monocytes and eosinophils (Fig. 7F, G, H and I). Also without excess corticosterone exposure, treatment with CORT125281 did not alter immune counts (Supplementary Fig. 4D). Subchronic corticosterone exposure induced hyperinsulinemia, and this was partially prevented by CORT125281 and fully prevented by mifepristone treatment, resulting in improved glucose levels and HOMA-IR (Fig. 7J, K and L). Both mifepristone and CORT125281 seemed to modestly reduce corticosterone-induced hyperlipidaemia (Fig. 7M, N and O). Finally, we evaluated the expression of GR target genes *Fkbp5*, *Gilz*, *Sgk1* and *Mt2a* in liver, muscle, BAT, WAT and hippocampus upon subchronic corticosterone exposure. Mifepristone effectively prevented corticosterone-induced expression of several GR target genes (Fig. 8A, B, C, D and E) and reduced basal mRNA expression of some GR target genes (e.g. *Gilz* in liver, muscle and BAT; *Mt2a* in BAT). CORT125281 did not prevent the upregulation of GR target gene expression induced by subchronic corticosterone in any of the tissues (Fig. 8A, B, C, D and E). As insulin levels were reduced by CORT125281, we additionally evaluated expression of insulin-dependent target genes *G6p* and *Pepck* in liver, glucose transporter 4 (*Glut4*) and lipoprotein lipase (*Lpl*) in WAT and expression of *Glut4* and glycogen synthase kinase 3b (*Gsk3b*) in muscle (Fig. 8A, B and D). We observed no significant alterations in these genes. In conclusion, CORT125281 showed less GR antagonism than mifepristone, but did improve corticosterone-induced hyperinsulinemia.

## Discussion

In this study, we investigated the efficacy of the selective GR antagonist CORT125281 across several doses in various GC-sensitive tissues during acute and subchronic corticosterone exposure. In the acute setting, CORT125281 (at a high dose of 60 mg/kg/day) was fully efficacious as a GR antagonist in the liver, while in BAT and in muscle, corticosterone-induced expression of only some target genes was attenuated. In WAT and in the brain, no GR antagonism by CORT125281 was observed. Lower doses of CORT125281 revealed dose- and gene-specific GR antagonism in liver, while the antagonistic effects of CORT125281 on other tissues were completely lost. During subchronic corticosterone exposure, CORT125281 at 60 mg/kg/day partially restored corticosterone-induced hyperinsulinemia, but was inferior to a similar dose of mifepristone in reversing corticosterone effects on immune cells and the expression of GR target genes.

CORT125281 was only effective in a subset of tissues and not in the hippocampus and WAT. The lack of GR antagonism in the brain can be explained by low tissue concentrations (possibly due to poor blood-brain-barrier penetrance of the compound). In contrast, tissue levels of CORT125281 did not explain the observed tissue specificity in other tissues, as high compound levels in WAT did not result in GR antagonism. An alternative explanation is that particular corepressor proteins are required for effective GR inhibition by CORT125281 (Meijer *et al.* 2006, Zalachoras *et al.* 2013). However, CORT125281 only induced a few interactions with coregulator motifs. More specifically, the CORT125281-GR complex weakly bound the motifs of corepressors NCOR1 and 2, which are recruited by mifepristone to actively silence gene expression (Schoch *et al.* 2010). These corepressors may play a role in the tissue specificity, but are not intrinsically necessary for antagonism, given that, in the acute setting, CORT125281 did act as a full antagonist on corticosterone-induced transcription in the liver. For other motifs that predominantly belong to transcriptional coactivators rather than corepressors, CORT125281 was weaker than mifepristone, and this may relate to the very low partial agonism of CORT125281 that has previously been observed in reporter assays (Kroon *et al.* 2018). It remains undetermined whether differential coregulator recruitment underlies the tissue-specific actions of CORT125281, although it may account for some of the functional disparities between the two compounds.

CORT125281 treatment had metabolic benefit, as it significantly lowered corticosterone-induced hyperinsulinemia. We were unable to elucidate the responsible mechanism. It is possible that CORT125281 directly interferes with β-cell function, given that GC can directly act on β-cells to stimulate insulin and glucagon release (Delaunay *et al.* 1997, Rafacho *et al.* 2014). Although CORT125281 treatment did not alter expression of insulin-dependent target genes in liver, WAT and muscle, post-translational modifications and cellular localization (which play a crucial role in *Glut4* regulation (Bryant *et al.* 2002)) may have been affected by COR125281.

COR125281 was less effective than RU486 in antagonizing the effects of subchronic corticosterone exposure. This difference may be explained by the different half maximal inhibitory concentrations of the two antagonists, with IC_50_ values of 43 nM for mifepristone and 427 nM for CORT125281 (Lahteenmaki *et al.* 1987, Kroon *et al.* 2018). Therefore, the dose of CORT125281 likely was inadequate to fully antagonize GR upon subchronic corticosterone exposure.

At a CORT125281 dose that was sufficient for effective antagonism, HPA-axis activity was relatively unaffected during basal and stressed conditions. Acute GR antagonism with mifepristone is known to further increase stimulated GC levels due to disinhibition of the HPA-axis (Gaillard *et al.* 1984, De Kloet *et al.* 1988), while chronic administration can lead to suppression of the HPA-axis (van den Heuvel *et al.* 2016, Kroon *et al.* 2018, Dalm *et al.* 2019). In our study, we evaluated CORT125281 only in a continuous and not in an acute treatment regimen, which may have allowed for adaptation of HPA-axis responsiveness. Given that during excess corticosterone exposure CORT125281 did not antagonize GR in the brain, including at the PVN level, we hypothesize that the negative feedback loop mediated via the PVN may still be intact and was dominant over the negative feedback exerted at the pituitary, confirming earlier findings (de Kloet *et al.* 1977). The pituitary expresses transcortin-like molecules that bind and neutralize local corticosterone levels thereby dampening pituitary negative feedback (de Kloet *et al.* 1977). Previously published work demonstrates an important role of GR in the PVN for adequate negative feedback on the HPA-axis (Laryea *et al.* 2013), while disruption of GR in the level of the pituitary only has a modest effect on HPA-axis activity in the context of fluctuations of endogenous glucocorticoids (Schmidt *et al.* 2009). In addition, we observed that CORT125281 increased both *Fkbp5* hnRNA and *Nr3c2* mRNA levels in the hippocampus. We hypothesize that *Fkbp5* expression followed *Nr3c2* expression, as we previously showed that FKBP5 is likely a direct MR target gene in the hippocampus (van Weert *et al.* 2019).

In conclusion, CORT125281 is a peripheral GR antagonist acting in a tissue- and dose-dependent manner that does not affect basal or stress-induced HPA-axis activity. Tailored dosing of CORT125281 may allow tissue-specific GR inhibition by bypassing effects on other target tissues.

## Supplementary Material

Supplementary Table 1

Sup. Fig. 1: The effect of CORT125281 treatment on body weight, food intake and GR target gene expression. Mice received vehicle or 60 mg/kg/day CORT125281 via the diet for 6 days. A-C) Body weight and food intake was unaffected by CORT125281 treatment. CORT125281 reduced expression of GR target genes Gilz and G6pc in the D) liver, but did not affect target gene expression in E) quadriceps muscle and F) gonadal WAT. D) In BAT, CORT125281 increased expression of Cd36, while in E) hippocampus Sgk1 and Mt2a were modestly decreased. Values are means ± SEM of N=7 mice per group. Statistical significance was calculated using independent sample t test. * p<0.05.

Sup. Fig. 2: CORT125281 did not affect body weight or food intake. Mice received vehicle or 6-20 mg/kg/day CORT125281 via the diet for 6 days. A-C) CORT125281 did not influence body weight (BW) or food intake. Values are means ± SEM of N=7-8 mice per group. Statistical significance was calculated using a one-way ANOVA with Tukey’s multiple comparisons test.

Sup. Fig. 3: CORT125281 increases GR mRNA expression in the anterior pituitary. Mice received vehicle or 20 mg/kg/day CORT125281 for 6 days and received a vehicle or corticosterone injection 1 hour prior to killing. A-C) CORT125281 increased GR mRNA expression (green) in corticotropic (red) and non-corticotropic cells. D) GR mRNA expression was most abundant in POMC-positive corticotropes. Values are means ± SEM of N=7-8 mice per group. Statistical significance was calculated using linear mixed models. * p<0.05, ***p<0.001.

Sup. Fig. 4: CORT125281 does not influence immune cell counts. Mice received vehicle or 60 mg/kg/day CORT125281 via the diet for 6 days. A-D) CORT125281 did not affect the number of total white blood cells (WBC), lymphocytes, monocytes and eosinophils. Values are means ± SEM of N=7 mice per group. Statistical significance was calculated using independent sample t test.

## Declaration of interest

H H is an employee of Corcept Therapeutics, a pharmaceutical company that develops glucocorticoid receptor antagonists, including CORT125281. O M receives funding from Corcept Therapeutics. The other authors have nothing to disclose.

## Funding

This study was partially funded by Corcept Therapeutics. L K was funded with a grant by the Board of Directors of Leiden University Medical Center.

## References

[bib1] Bryant NJ Govers R James DE 2002 Regulated transport of the glucose transporter GLUT4. Nature Reviews: Molecular Cell Biology 3 267–277. (10.1038/nrm782) 11994746

[bib2] Cuevas-Ramos D Lim DST Fleseriu M 2016 Update on medical treatment for Cushing’s disease. Clinical Diabetes and Endocrinology 2 16 (10.1186/s40842-016-0033-9) 28702250 PMC5471955

[bib3] Dalm S Karssen AM Meijer OC Belanoff JK De Kloet ER 2019 Resetting the stress system with a mifepristone challenge. Cellular and Molecular Neurobiology 39 503–522. (10.1007/s10571-018-0614-5) 30173378 PMC6469632

[bib4] De Kloet ER Burbach P Mulder GH 1977 Localization and role of transcortin-like molecules in the anterior pituitary. Molecular and Cellular Endocrinology 7 261–273. (10.1016/0303-7207(77)90058-2) 873047

[bib5] De Kloet ER De Kock S Schild V Veldhuis HD 1988 Antiglucocorticoid RU 38486 attenuates retention of a behaviour and disinhibits the hypothalamic-pituitary adrenal axis at different brain sites. Neuroendocrinology 47 109–115. (10.1159/000124900) 3344063

[bib6] Delaunay F Khan A Cintra A Davani B Ling ZC Andersson A Ostenson CG Gustafsson J Efendic S Okret S 1997 Pancreatic beta cells are important targets for the diabetogenic effects of glucocorticoids. Journal of Clinical Investigation 100 2094–2098. (10.1172/JCI119743) 9329975 PMC508401

[bib7] Desmet SJ Dejager L Clarisse D Thommis J Melchers D Bastiaensen N Ruijtenbeek R Beck IM Libert C Houtman R, ***et al*** 2014 Cofactor profiling of the glucocorticoid receptor from a cellular environment. Methods in Molecular Biology 1204 83–94. (10.1007/978-1-4939-1346-6_8) 25182763

[bib8] Ferrau F Korbonits M 2015 Metabolic comorbidities in Cushing’s syndrome. European Journal of Endocrinology 173 M133–M157. (10.1530/EJE-15-0354) 26060052

[bib9] Gaillard RC Riondel A Muller AF Herrmann W Baulieu EE 1984 RU 486: a steroid with antiglucocorticosteroid activity that only disinhibits the human pituitary-adrenal system at a specific time of day. PNAS 81 3879–3882. (10.1073/pnas.81.12.3879) 6328529 PMC345325

[bib10] Havel PJ Busch BL Curry DL Johnson PR Dallman MF Stern JS 1996 Predominately glucocorticoid agonist actions of RU-486 in young specific-pathogen-free Zucker rats. American Journal of Physiology 271 R710–R717. (10.1152/ajpregu.1996.271.3.R710) 8853395

[bib11] Herman JP Mcklveen JM Ghosal S Kopp B Wulsin A Makinson R Scheimann J Myers B 2016 Regulation of the hypothalamic-pituitary-adrenocortical stress response. Comprehensive Physiology 6 603–621. (10.1002/cphy.c150015) 27065163 PMC4867107

[bib12] Hunt HJ Belanoff JK Walters I Gourdet B Thomas J Barton N Unitt J Phillips T Swift D Eaton E 2017 Identification of the clinical candidate (R)-(1-(4-fluorophenyl)-6-((1-methyl-1H-pyrazol-4-yl)sulfonyl)-4,4a,5,6,7,8-hexah ydro-1H-pyrazolo[3,4-g]isoquinolin-4a-yl)(4-(trifluoromethyl)pyridin-2-yl)methanone (CORT125134): a selective glucocorticoid receptor (GR) antagonist. Journal of Medicinal Chemistry 60 3405–3421. (10.1021/acs.jmedchem.7b00162) 28368581

[bib13] Imai E Stromstedt PE Quinn PG Carlstedt-Duke J Gustafsson JA Granner DK 1990 Characterization of a complex glucocorticoid response unit in the phosphoenolpyruvate carboxykinase gene. Molecular and Cellular Biology 10 4712–4719. (10.1128/mcb.10.9.4712) 2388623 PMC361067

[bib14] Jung-Testas I Baulieu EE 1983 Inhibition of glucocorticosteroid action in cultured L-929 mouse fibroblasts by RU 486, a new anti-glucocorticosteroid of high affinity for the glucocorticosteroid receptor. Experimental Cell Research 147 177–182. (10.1016/0014-4827(83)90282-3) 6617759

[bib15] Karssen AM Meijer OC Van Der Sandt IC Lucassen PJ De Lange EC De Boer AG De Kloet ER 2001 Multidrug resistance P-glycoprotein hampers the access of cortisol but not of corticosterone to mouse and human brain. Endocrinology 142 2686–2694. (10.1210/endo.142.6.8213) 11356720

[bib16] Kloosterboer HJ Deckers GH De Gooyer ME Dijkema R Orlemans EO Schoonen WG 1995 Pharmacological properties of a new selective antiprogestagen: Org 33628. Annals of the New York Academy of Sciences 761 192–201. (10.1111/j.1749-6632.1995.tb31379.x) 7625721

[bib17] Koppen A Houtman R Pijnenburg D Jeninga EH Ruijtenbeek R Kalkhoven E 2009 Nuclear receptor-coregulator interaction profiling identifies TRIP3 as a novel peroxisome proliferator-activated receptor gamma cofactor. Molecular and Cellular Proteomics 8 2212–2226. (10.1074/mcp.M900209-MCP200) 19596656 PMC2758751

[bib18] Kroon J Koorneef LL Van Den Heuvel JK Verzijl CRC Van De Velde NM Mol IM Sips HCM Hunt H Rensen PCN Meijer OC 2018 Selective glucocorticoid receptor antagonist CORT125281 activates brown adipose tissue and alters lipid distribution in male mice. Endocrinology 159 535–546. (10.1210/en.2017-00512) 28938459

[bib19] Lahteenmaki P Heikinheimo O Croxatto H Spitz I Shoupe D Birgerson L Luukkainen T 1987 Pharmacokinetics and metabolism of RU 486. Journal of Steroid Biochemistry 27 859–863. (10.1016/0022-4731(87)90160-9) 3695508

[bib20] Laryea G Schutz G Muglia LJ 2013 Disrupting hypothalamic glucocorticoid receptors causes HPA axis hyperactivity and excess adiposity. Molecular Endocrinology 27 1655–1665. (10.1210/me.2013-1187) 23979842 PMC4061381

[bib21] Meijer OC Van Der Laan S Lachize S Steenbergen PJ De Kloet ER 2006 Steroid receptor coregulator diversity: what can it mean for the stressed brain? Neuroscience 138 891–899. (10.1016/j.neuroscience.2005.07.004) 16310313

[bib22] Mifsud KR Reul JM 2016 Acute stress enhances heterodimerization and binding of corticosteroid receptors at glucocorticoid target genes in the hippocampus. PNAS 113 11336–11341. (10.1073/pnas.1605246113) 27655894 PMC5056104

[bib23] Monczor F Chatzopoulou A Zappia CD Houtman R Meijer OC Fitzsimons CP 2019 A model of glucocorticoid receptor interaction with coregulators predicts transcriptional regulation of target genes. Frontiers in Pharmacology 10 214 (10.3389/fphar.2019.00214) 30930776 PMC6425864

[bib24] Morgan SA Hassan-Smith ZK Lavery GG 2016 MECHANISMS IN ENDOCRINOLOGY: Tissue-specific activation of cortisol in Cushing’s syndrome. European Journal of Endocrinology 175 R83–R89. (10.1530/EJE-15-1237) 26957494

[bib25] Nixon M Mackenzie SD Taylor AI Homer NZ Livingstone DE Mouras R Morgan RA Mole DJ Stimson RH Reynolds RM, ***et al*** 2016 ABCC1 confers tissue-specific sensitivity to cortisol versus corticosterone: a rationale for safer glucocorticoid replacement therapy. Science Translational Medicine 8 352ra109 (10.1126/scitranslmed.aaf9074) 27535620

[bib26] Rafacho A Ortsater H Nadal A Quesada I 2014 Glucocorticoid treatment and endocrine pancreas function: implications for glucose homeostasis, insulin resistance and diabetes. Journal of Endocrinology 223 R49–R62. (10.1530/JOE-14-0373) 25271217

[bib27] Schmidt MV Sterlemann V Wagner K Niederleitner B Ganea K Liebl C Deussing JM Berger S Schutz G Holsboer F, ***et al*** 2009 Postnatal glucocorticoid excess due to pituitary glucocorticoid receptor deficiency: differential short- and long-term consequences. Endocrinology 150 2709–2716. (10.1210/en.2008-1211) 19213843

[bib28] Schoch GA D’arcy B Stihle M Burger D Bar D Benz J Thoma R Ruf A 2010 Molecular switch in the glucocorticoid receptor: active and passive antagonist conformations. Journal of Molecular Biology 395 568–577. (10.1016/j.jmb.2009.11.011) 19913032

[bib29] Shimizu N Yoshikawa N Ito N Maruyama T Suzuki Y Takeda S Nakae J Tagata Y Nishitani S Takehana K, ***et al*** 2011 Crosstalk between glucocorticoid receptor and nutritional sensor mTOR in skeletal muscle. Cell Metabolism 13 170–182. (10.1016/j.cmet.2011.01.001) 21284984

[bib30] Van Den Heuvel JK Boon MR Van Hengel I Peschier-Van Der Put E Van Beek L Van Harmelen V Van Dijk KW Pereira AM Hunt H Belanoff JK, ***et al*** 2016 Identification of a selective glucocorticoid receptor modulator that prevents both diet-induced obesity and inflammation. British Journal of Pharmacology 173 1793–1804. (10.1111/bph.13477) 26990179 PMC4867737

[bib31] Van Weert LTCM Buurstede JC Sips HCM Vettorazzi S Mol IM Hartmann J Prekovic S Zwart W Schmidt MV Roozendaal B, ***et al*** 2019 Identification of mineralocorticoid receptor target genes in the mouse hippocampus. Journal of Neuroendocrinology 31 e12735 (10.1111/jne.12735) 31121060 PMC6771480

[bib32] Vander Kooi BT Onuma H Oeser JK Svitek CA Allen SR Vander Kooi CW Chazin WJ O’Brien RM 2005 The glucose-6-phosphatase catalytic subunit gene promoter contains both positive and negative glucocorticoid response elements. Molecular Endocrinology 19 3001–3022. (10.1210/me.2004-0497) 16037130

[bib33] Waddell DS Baehr LM Van Den Brandt J Johnsen SA Reichardt HM Furlow JD Bodine SC 2008 The glucocorticoid receptor and FOXO1 synergistically activate the skeletal muscle atrophy-associated MuRF1 gene. American Journal of Physiology: Endocrinology and Metabolism 295 E785–E797. (10.1152/ajpendo.00646.2007) 18612045 PMC2652500

[bib34] Zalachoras I Houtman R Atucha E Devos R Tijssen AM Hu P Lockey PM Datson NA Belanoff JK Lucassen PJ, ***et al*** 2013 Differential targeting of brain stress circuits with a selective glucocorticoid receptor modulator. PNAS 110 7910–7915. (10.1073/pnas.1219411110) 23613579 PMC3651427

