# Supplementary material for: The selective glucocorticoid receptor antagonist CORT125281 has tissue-specific activity
Source: J Endocrinol. 2020 May 4;246(1):79–92. doi: 10.1530/JOE-19-0486 (PMC7274539; doi:10.1530/JOE-19-0486)
Supplement: Supplementary Table 1 [file supplementary_table_1.pdf]

Gene	Primer fw	Primer rev
Angptl4	GGAAAGAGGCTTCCCAAGAT	TCCCAGGACTGGTTGAAGTC
Fas	GCGCTCCTCGCTTGTCGTCT	TAGAGCCCAGCCTTCCATCTCCTG
Fat/Cd36	GCAAAGAACAGCAGCAAAATC	CAGTGAAGGCTCAAAGATGG
Fkbp5	GCCGACTGTGTGTGTAATGC	CACAATACGCACTTGGGAGA
G6pc	TCCTCTTTCCCATCTGGTTC	TATACACCTGCTGCGCCCAT
Gilz	TGGCCCTAGACAACAAGATTGAGC	CCACCTCCTCTCTCACAGCAT
Glut4	CAGCGCCTGAGTCTTTTCTT	GGCATTGATAACCCCAATGT
Gsk3b	AAGGAAGGAAAAGGTGATTCAAGA	TGCTGCCATCTTTATCTCTGCTA
Lpl	CCCTAAGGACCCCTGAAGAC	GGCCCGATACAACCAGTCTA
Mt2a	ACGTCCTGAGTACCTTCTCCT	GGAGGTGCACTTGCAGTTCTTG
Murf1	TGTGCAAGGAACACGAAGA	CCAGCATGGAGATGCAGTT
Pepck	ATCTTTGGTGGCCGTAGACCT	GCCAGTGGGCCAGGTATTT
Redd1	GCCGGAGGAAGACTCCTCA	CATCAGGTTGGCACACAGG
Sgk1	AGAGGCTGGGTGCCAAGGAT	CACTGGGCCCGCTCACATTT
Ucp1	TCAGGATTGGCCTCTACGAC	TGCATTCTGACCTTCACGAC
