# Supplementary figures and images for: The selective glucocorticoid receptor antagonist CORT125281 has tissue-specific activity
Source: J Endocrinol. 2020 May 4;246(1):79–92. doi: 10.1530/JOE-19-0486 (PMC7274539; doi:10.1530/JOE-19-0486)

**A**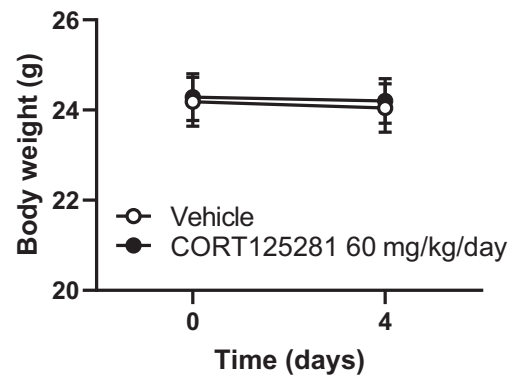**B**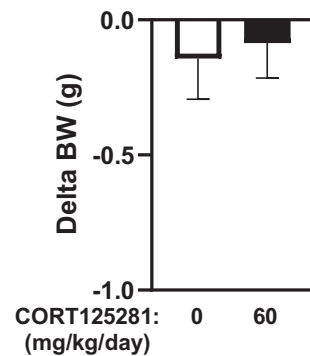**C**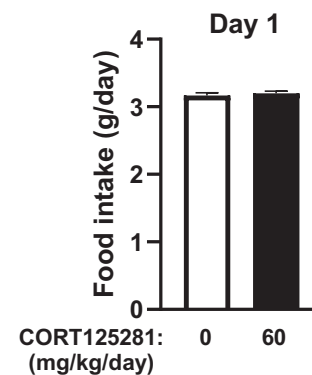**D**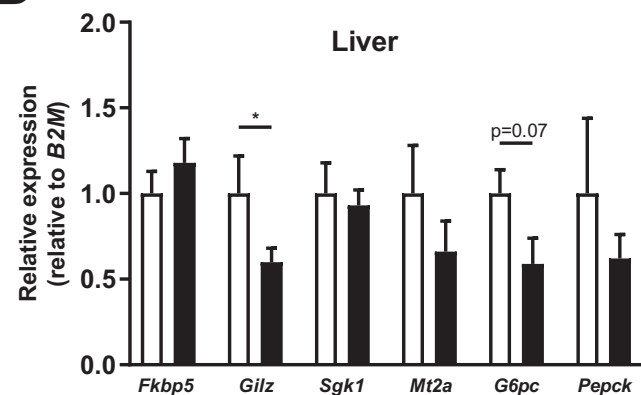**E**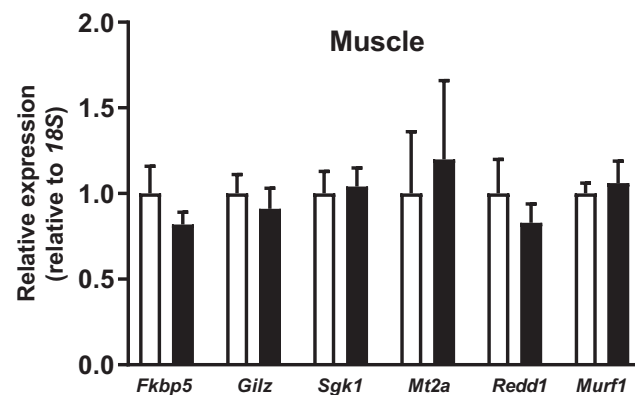**F**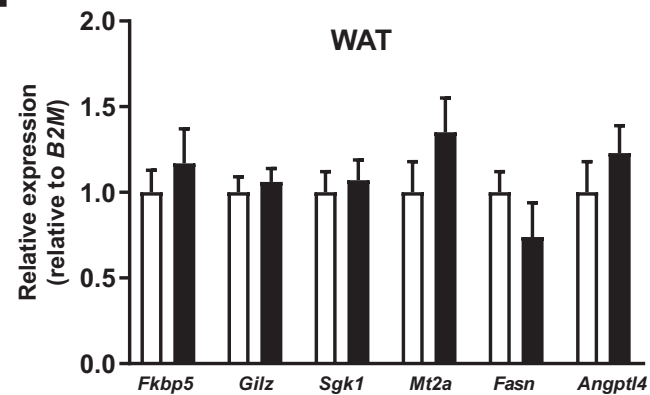**G**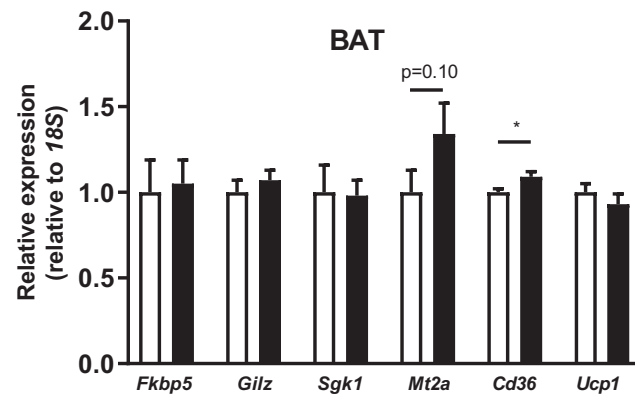**H**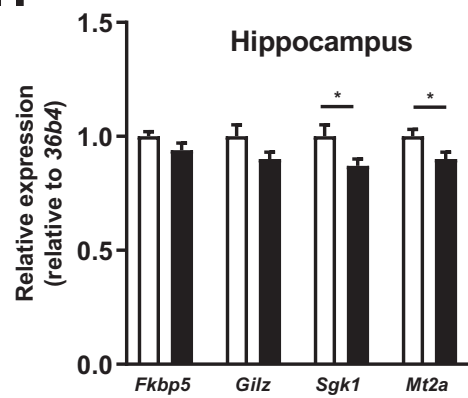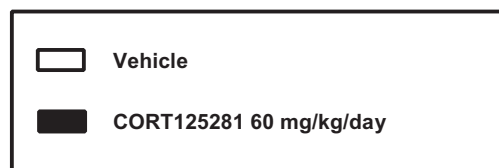

Supplement: Sup. Fig. 1: The effect of CORT125281 treatment on body weight, food intake and GR target gene expression. Mice received vehicle or 60 mg/kg/day CORT125281 via the diet for 6 days. A-C) Body weight and food intake was unaffected by CORT125281 treatment. CORT125281 reduced expression of GR target gen [file supplementary_figure_1.pdf]

**A**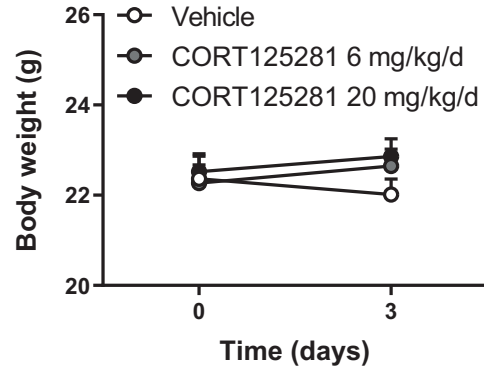**B**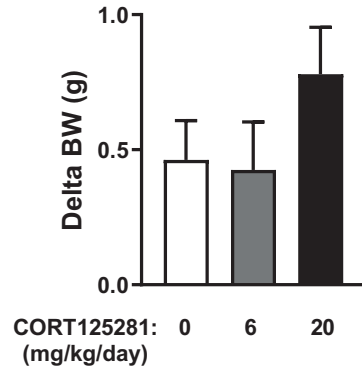**C**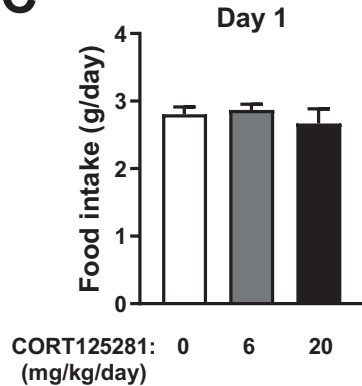

Supplement: Sup. Fig. 2: CORT125281 did not affect body weight or food intake. Mice received vehicle or 6-20 mg/kg/day CORT125281 via the diet for 6 days. A-C) CORT125281 did not influence body weight (BW) or food intake. Values are means ± SEM of N=7-8 mice per group. Statistical significance was calculated us [file supplementary_figure_2.pdf]

**A**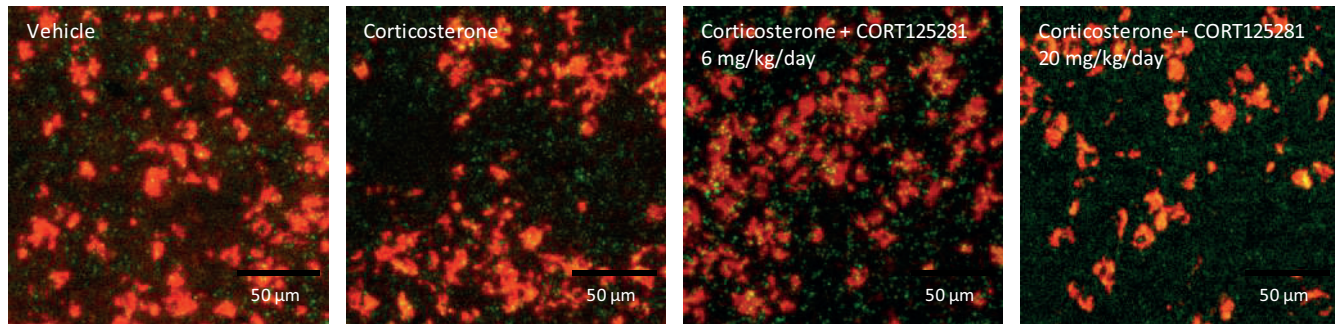**B**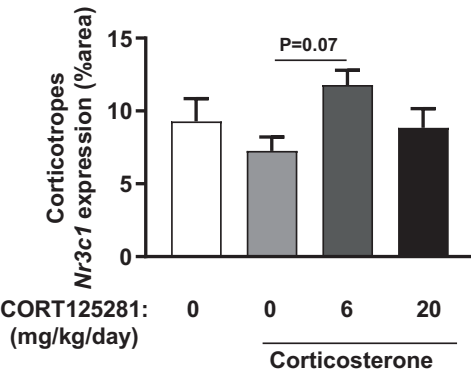**C**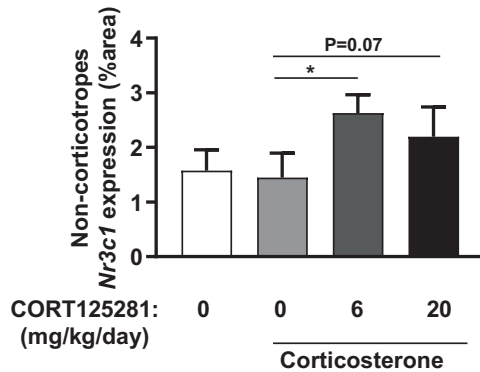**D**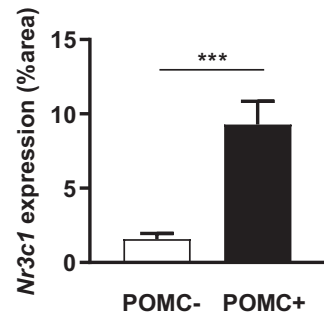

Supplement: Sup. Fig. 3: CORT125281 increases GR mRNA expression in the anterior pituitary. Mice received vehicle or 20 mg/kg/day CORT125281 for 6 days and received a vehicle or corticosterone injection 1 hour prior to killing. A-C) CORT125281 increased GR mRNA expression (green) in corticotropic (red) and non- [file supplementary_figure_3.pdf]

**A**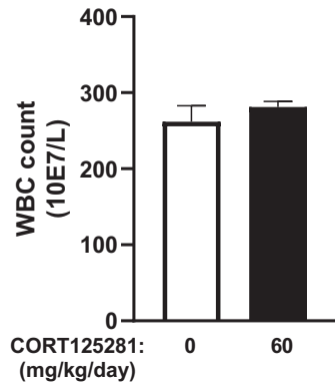**B**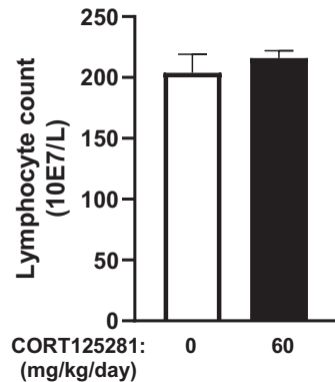**C**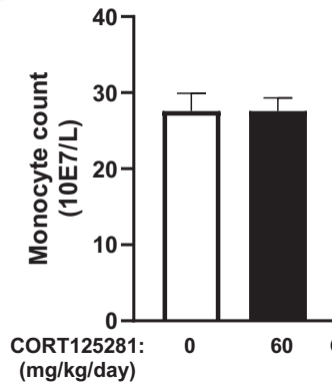**D**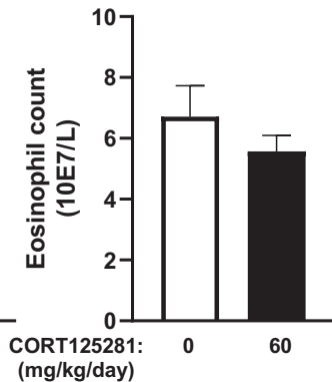

Supplement: Sup. Fig. 4: CORT125281 does not influence immune cell counts. Mice received vehicle or 60 mg/kg/day CORT125281 via the diet for 6 days. A-D) CORT125281 did not affect the number of total white blood cells (WBC), lymphocytes, monocytes and eosinophils. Values are means ± SEM of N=7 mice per group. S [file supplementary_figure_4.pdf]
